# Transient Increases in Alpha Power Relative to Healthy Reference Ranges in Awake Piglets After Repeated Rapid Head Rotations

**DOI:** 10.3390/biomedicines12112460

**Published:** 2024-10-26

**Authors:** Anna Oeur, William H. Torp, Susan S. Margulies

**Affiliations:** Wallace H. Coulter Department of Biomedical Engineering, Emory University and Georgia Institute of Technology, Atlanta, GA 30332, USA; anna.oeur@emory.edu (A.O.); wtorp3@gatech.edu (W.H.T.)

**Keywords:** alpha power, beta power, EEG, concussion, porcine, traumatic brain injury, resting state

## Abstract

**Background/Objectives**: Sports-related concussions are a main cause of cognitive dysfunction and somatic complaints, particularly in youth. While the majority of concussion symptoms resolve within one week, cognitive effects may persist. In this study, we sought to study changes to cognition within this acute time frame. **Methods**: In this current study, we use an established swine model of traumatic brain injury (TBI) to study the effects of single and repeated head rotations on resting-state electroencephalography (rs-EEG) in awake piglets in the acute (within 7 days) time period after injury. We studied both healthy and experimental groups to (1) establish healthy reference ranges (RRs; N = 23) for one-minute rs-EEG in awake piglets, (2) compare the effects of single (N = 12) and repeated head rotations (N = 13) on rs-EEG, and (3) examine the acute time course (pre-injury and days 1, 4, and 7 post-injury) in animals administered single and repeated head rotations. EEG data were Fourier transformed, and total (1–30 Hz) and relative power in the alpha (8–12 Hz), beta (16.5–25 Hz), delta (1–4 Hz), and theta (4–7.5 Hz) bands were analyzed. **Results**: Total power and relative alpha, beta, delta, and theta power were consistent measures across days in healthy animals. We found a significant and transient increase in relative alpha power after repeated injury on day 1 in all regions and a rise above the healthy RR in the frontal and left temporal regions. **Conclusions**: Future studies will expand the study duration to investigate and inform clinical prognoses from acute measurements of rs-EEG.

## 1. Introduction

Between 1.6 and 3.5 million people sustain a mild traumatic brain injury (mTBI) annually [1]. Data from the National Electronic Injury Surveillance System—All Injury Program from 2001–2012 reported that 70% of patients seeking care for a sports-related mTBI at a US emergency department were 19 years old or younger [2]. The pediatric population is particularly susceptible to sustaining mTBIs and suffering from their sequelae due to their high participation rates in organized sports and recreation combined with the effect of injury on the developing brain [3,4].

Traumatic brain injury can be caused by a biomechanical force imparted to the head to induce a rapid acceleration/deceleration on the intracranial components, resulting in focal and diffuse stresses and strains [5]. The severity and types of brain injury outcomes are related to the nature of biomechanical loading (direction and magnitude), with predominantly linear accelerations causing focal injuries (i.e., contusions, skull fractures), and rotational accelerations causing diffuse brain injuries (i.e., diffuse axonal injury, concussion) [6]. Secondary injuries may also ensue after the traumatic event, causing a disruption to the pathophysiology of the brain [7]. Stretching and shearing of the neuronal cells initiate neuroinflammatory mechanisms and mitochondrial dysfunctions that lead to imbalances of intra-and extracellular ions that cause functional impairments [8].

Early experimental TBI research illustrated suppressions of electrical activity directly after the moment of impact and the degree of these suppressions related to the severity of the injury [9,10]. Neural oscillations are the rhythmic firings of groups of neurons in the brain that coordinate the communication between these regions to carry out various cognitive tasks [11]. One of the most common methods for quantifying the electrical activity of the brain is resting-state electroencephalography (rs-EEG), which captures neural oscillations in the alert and awake individual and can provide insight into baseline intrinsic cognitive processes without an extrinsic task or stimuli. In the healthy and awake individual, rs-EEG data captured on the surface of the scalp appear as small rhythmic fluctuations in electrical activity. Spectral analyses can then be conducted to quantify the patterns of neural oscillations to determine the amount (as reflected by power) of the signal that is oscillating between 8 and 12 Hz (alpha band), 16.5 and 25 Hz (beta), 1 and 3.5 Hz (delta), and 4 and 7.5 Hz (theta). Each band has been associated with different cognitive processes [12,13]. Alpha activity is observed in an awake and eyes-closed person in the occipital, frontal, and parietal regions, with this activity largely being attenuated once the eyes are opened [14,15]. Additionally, with eyes open, alpha activity is associated with increased attention and task engagement [16]. Beta activity is seen during active thinking and corresponds to activity in the sensorimotor cortex [14,15]. An increase in the slower-frequency bands, theta and delta, are both associated with drowsiness in awake persons [15]. Generally, an increase in theta is associated with memory tasks, emotional distress, and other neurological conditions [14,16,17].

Rs-EEG is commonly used to study the effects of TBI on neural function and has been shown to be able to detect electrophysiological abnormalities [18]. Next, results from systematic reviews focused on mTBI are summarized to present generalized findings on frequency band increases or decreases after injury. A recent systematic review of acute/subacute (≤28 days) or chronic (>28 days) EEG responses of sports-related concussions (SRCs) in adolescent athletes revealed differences between injured and control groups [19], with alterations associated with information flow, connectivity, and computed composite scores in the acute/subacute time period. The chronic stage revealed increased delta and theta and decreased alpha and beta power [19]. A more recent systematic review of 31 papers that examined a broad range of traumatic brain injury severity and age range (children to adults) also found an increase in slow waves (delta and theta bands) and a subsequent decrease in fast alpha and beta waves [20]. Conley et al. (2019) summarized 16 studies on rs-EEG after SRCs, and the theta band was found to be most often different between injured and control groups, but findings were inconsistent as to whether it increased or decreased [21]. One possible reason for inconsistent theta changes is the large age range (15–50 years old) of the participants in the studies because age has been shown to influence brain oscillations [22]. We conclude that the use of rs-EEG measures to distinguish between mTBI and healthy subjects is influenced by many factors and is not limited to the timing of measurement after the injury, age of the subject, and injury mechanism (sports-related, motor vehicle accident, or military/combat-related).

Pre-clinical animal studies offer an idealized platform to study TBI as these models permit the control of many factors. Among animals, swine models are commonly used to explore biomarkers for mTBI [23] as they have relatively large brains (in contrast to rodents) and well-defined gyri and sulci features that allow for the use of human-designed EEG systems during studies, which can help to enhance translatability between clinical and pre-clinical research [24]. The objective of this study is to examine the effect of biomechanical loads on changes in cognition as measured by rs-EEG and the acute time course after injury. In this current study, we use an established rapid non-impact rotation (RNR) swine model of TBI [25] to study the effects of single and repeated head rotations on rs-EEG activity in awake piglets in the acute (within 7 days) time period after injury. To overcome the inter-subject variability associated with EEG measures, we studied both healthy and experimental groups. First, we studied healthy animals to establish healthy reference ranges for rs-EEG activity in awake piglets. Then, in a separate group, animals allocated to sham anesthesia or single or repeated head rotations were studied, and awake rs-EEG measures were captured pre-injury and on days 1, 4, and 7 after injury. We compare these experimental animals to the healthy reference ranges to determine the proportion of animals that fall outside of these ranges per study day. Lastly, we examine the acute time course (within 7 days) using rs-EEG measures for sham, single, and repeated injury animals.

## 2. Materials and Methods

To examine changes to rs-EEG following acute TBI, we used 4-week-old female Yorkshire piglets for this study. Animals were allocated to healthy, validation, or experimental groups. Healthy and validation animals were studied for up to 3 days to collect 1-min awake rs-EEG. Experimental animals were further divided into groups of anesthetized sham animals or animals that received a single rotation or repeated head rotations. The kinematic load and frequency of these head rotations were informed via cross-species scaling from human data to inform piglet kinematics. For the experimental animals, 1 min of RS-EEG was captured before injury to establish a baseline value, and it was captured again on days 1, 4, and 7 following injury to investigate the effect of head rotations on rs-EEG. Power values for alpha, beta, delta, and theta frequency bands were extracted and grouped into front, right, and left temporal regions for all animals.

### 2.1. Animal Subjects

A total of seventy-six 4-week-old female Yorkshire piglets (Sus scrofa) were included in this study and were received from Palmetto Research Swine (Reevesville, SC, USA), Oak Hill Genetics (Franklin County, IL, USA), or Premier BioSource (Romona, CA, USA). Animals were housed in groups of 2–4 littermates, on a 12-h light and 12-h dark cycle, and permitted to eat food pellets (LabDiet 5080, St. Louis, MO, USA) and drink water ad libitum. Animals were acclimated to research staff and data collection equipment for a minimum of two 30-min sessions when they were taken into the data collection room, placed in a Panepinto sling with the electroencephalography (EEG) net on their head, and closely monitored and supported by research staff. After acclimation, all data collection sessions were performed in the same room and with the same staff and equipment.

Of the seventy-six-animal cohort, twenty-three healthy animals were studied to establish healthy reference ranges, and data from eighteen additional healthy animals were used for validation. The remaining 35 animals were designated to one of three experimental groups: anesthetized shams (N = 10) or single (N = 12; sRNR) or repeated, rapid, non-impact head rotations (N = 13; rRNR). Procedures were approved by the Institutional Animal Care and Use Committee (IACUC) at Emory University School of Medicine. Experiments were carried out in an Association for Assessment and Accreditation of Laboratory Animal Care (AAALAC)-accredited facility.

### 2.2. Rapid Non-Impact Rotational Injury (RNR)

The sham, sRNR, and rRNR cohorts of animals were sedated using an intramuscular injection of 4 mg/kg of Ketamine, 2 mg/kg of Xylazine, and 0.2 mg/kg of Midazolam and were anesthetized using inhalation of 1.5% isoflurane gas before the injury was administered. Proper depth of anesthesia was confirmed by a lack of response to a toe pinch. A bite plate was used to secure the animals to a HYGE device (HYGE Inc., Kittaning, PA, USA). The HYGE device translates linear motion of a pneumatic actuator, via a linkage and bite plate, into a controlled rapid angular rotation of the head [25]. The angular velocity of the animal’s head was recorded using angular transducers mounted to the side-arm linkage of the HYGE device (ARS-06, ATA Engineering, Inc., Herndon, VA or ARS Pro, DTS Inc., Seal Beach, CA, USA).

Piglet biomechanical load magnitudes and timing in this study were informed by our previous work [26], in which cross-species brain tissue strain-based scaling techniques were employed on a dataset of 267 measured soccer headers in instrumented high school soccer. The soccer header impacts utilized occurred primarily in the front, with the dominant head rotation occurring in the sagittal plane. Load velocities and accelerations associated with the 50th and 90th percentile maximal tissue deformations were employed, scaled to the piglet. The HYGE device represents a header as a sudden sagittal acceleration–deceleration of the head, replicating ball-to-head contact kinematics in the absence of any skull deformation, fracture, or cortical contusion, which are rarely associated with typical ball-to-head contact. Briefly, the sRNR group received one rotation (angular velocity approximately 100 rad/s), and the rRNR group received the same rotation followed by four lower-velocity rotations (angular velocity approximately 60 rad/s), separated by 8.00 ± 0.03 min. The rRNR group received 5 rotations separated by 8 min to match the 4 to 6 impacts separated by 8 min observed in male and female high school soccer data, previously studied [27]. The kinematics were generated from 267 video-identified frontal soccer headers resulting in sagittal head loading [27] that were scaled to the piglet via a finite element model (FEM) of a human brain to generate the 50th (medium) and 90th (high) percentile maximal axonal strain (MAS), and the matching MAS was calculated [28,29] in a piglet FEM to determine the target angular velocity and acceleration that creates the same strained-based deformation in the piglet. The measured angular velocity and estimated acceleration are reported in Table 1.

### 2.3. EEG Data Acquisition

EEG data were collected using a custom-designed 32-electrode Hydrocel Geodesic Sensor Net (GSN) with a sampling rate of 1000 Hz (Electric Geodesics Inc., EGI, Eugene, OR, USA). The electrode net is an elastomer structure with embedded chambers containing a single electrode and sponge, with room for eyes and ears, and an adjustable chinstrap to ensure a close fit. A computer cart with a Hypertronics connector arm links the 32-electrode net to the Net Amps 400 amplifier (Electric Geodesics Inc., EGI, Eugene, OR, USA) and to a MacBook Pro laptop with NetStation Version 5.4.2 software (Electric Geodesics Inc., EGI, Eugene, OR, USA) for EEG data acquisition.

Prior to recording data, the EEG net was soaked in a solution of baby shampoo (5 mL) and potassium chloride (10 mL) mixed in 1 L of water for at least 5 min. The EEG net was then applied to the surface of the animal’s head, and the electrical impedance for all electrodes was checked to be below 1 kΩ. A light nylon stocking with holes cut out for ears and eyes was fitted over the electrode net to maintain scalp contact throughout data collection. The rs-EEG data were captured in awake piglets for 1 min in a well-ventilated, quiet, and brightly lit room. Healthy animals had data collected on three non-consecutive days, and experimental animals had data collected at pre-injury and 1, 4, and 7 days post-injury time points.

### 2.4. EEG Data Processing

**Figure 1:**
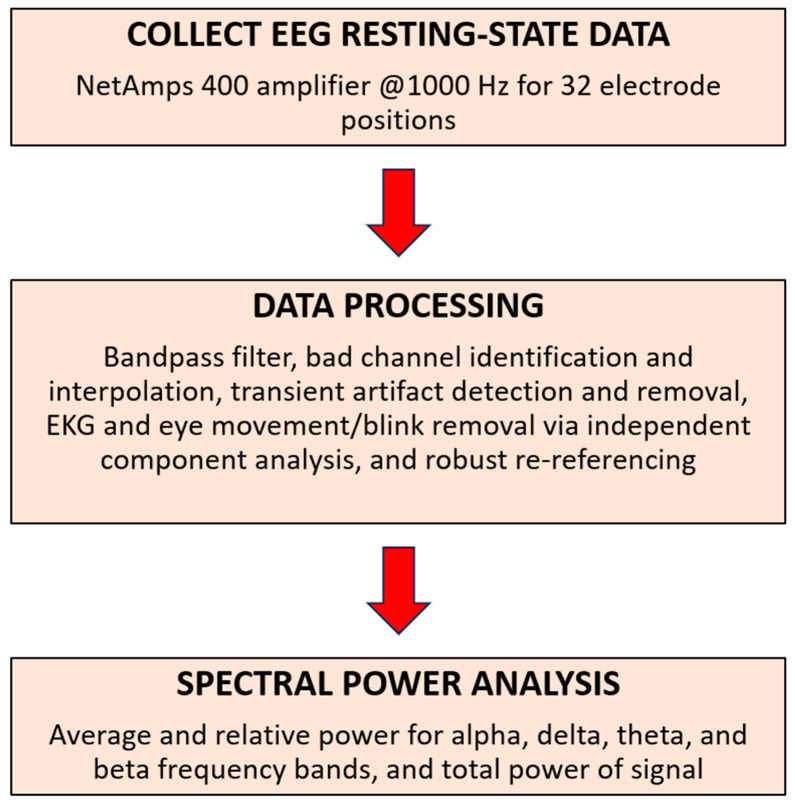
Flow chart for resting-state EEG data acquisition, pre-processing, and spectral analysis.

EEG pre-processing was performed in MATLAB Version 2023b (The MathWorks, Inc., Natick, MA, USA) and the FieldTrip toolbox [30] to import and pre-process EEG data, remove bad channels and transient artifacts, and to calculate electrode-level power spectra (Figure 1).

In FieldTrip, EEG data were filtered between 1 and 32 Hz using a 2000th-order, two-sided, finite impulse response bandpass filter with a hamming window [31]. The first and last 10 s of data were removed to eliminate noise from starting and ending the recording. The data were visually inspected on a scale from −75 to + 75 µV. Bad channels were identified as channels with large amplitude artifacts persistent during the entire length of the recording (>100 µV). These channels were interpolated using a weighted average of neighboring electrode activity determined through triangulation of the electrode layout. Transient artifacts, such as large movements of the animal’s head and muscle activity, were identified as large amplitude noise across many electrodes for a period of time within the recording. These sections of data were selected and removed from further analysis. Lastly, cardiac activity and blinks were removed using independent component analysis with the ‘fastica’ algorithm [32], and the fully cleaned data were then re-referenced across all channels using the average of all electrodes.

Power spectra were calculated using the ‘mtmfft’ method and a Hanning window in FieldTrip for alpha (8–12 Hz), beta (16.5–25 Hz), delta (1–3.5 Hz), and theta (4–7.5 Hz) frequency bands. The frequency precision within frequency bands was 0.25 Hz. Total power was calculated on a per-electrode basis by summing the power spectrum from 1 to 25 Hz. Relative power was calculated on a per-electrode basis by summing the power spectrum of a specific frequency band, dividing this frequency band-specific sum by the total power of that electrode, and then multiplying by 100%. For example, relative alpha power was calculated by summing the power spectrum of an electrode from 8 to 12 Hz, dividing the alpha-band sum by the total power for the same electrode, and then multiplying by 100%.

To determine regional values, total power in the signal and relative power in each frequency band were averaged across electrodes to calculate frontal (1,2,3,4,17,27), left temporal (5,11,13,15,23), right temporal (6,12,14,16,24), and occipital (9,10,19,20) regions on the scalp. A single power value for each region, day, and animal was used as input for statistical analysis.

### 2.5. Outlier Removal

Outlier analysis was completed by grouping animal data by injury group, study day, and region for each power band. Next, outliers were removed if the value was 3 times the interquartile range in each condition.

### 2.6. Healthy Reference Ranges

For animals in the healthy group (N = 23), a 1-way ANOVA for the effect of study day (1, 2, 3) was conducted to determine if rs-EEG differed by day in healthy animals. Day was not found to be significant for any power band (*p* < 0.05); therefore, we concluded that rs-EEG measures were consistent from day to day. Healthy reference ranges were established by averaging across days and calculating the 2.5th, 50th, and 97.5th percentiles for total power and relative alpha, beta, delta, and theta power (Table 2).

Data from the validation group, an additional 18 healthy animals with 1 or 2 days of data collected from male (N = 2) and female piglets, were used to validate each parameter and region of interest. The 2.5th and 97.5th healthy RRs (Table 2) were used to evaluate the percentage of validation animals (N = 18) that fell within these ranges; they are presented in Table 3. Each validation measurement was treated as a separate entry evaluated against the healthy RR. For example, if an animal had two measurements completed on separate days, each measurement was used as an independent entry in the evaluation. The percentages of validation animals within the RR (Table 3) for total power were all above our validation threshold of 75% for all regions except the occipital (61%); therefore, the occipital region was excluded from further analyses for relative power. All frequency bands for relative power had validation animals that were ≥79% in the RR in the front, left, and right temporal regions.

**Table 3:** Percentage of validations animals (N = 18) within the healthy RR (middle 95%) for total and relative power per region of interest.

Frequency Band	Frontal	Left Temporal	Right Temporal	Occipital
Total Power	93%	93%	89%	61%
Relative Alpha	93%	93%	96%	-
Relative Beta	96%	100%	96%	-
Relative Delta	89%	96%	96%	-
Relative Theta	93%	96%	79%	-

The established healthy RRs were used to evaluate the experimental groups (sham, single, and repeated) to determine the proportion of animals that were significantly within (or outside) the RRs for each study day. A Fischer’s Exact Test was used to test if there was a significant proportion of animals outside the healthy RRs per animal group for each study day, and a Cochran’s Q followed by a McNemar’s test determined if there was a significant proportion of animals outside the RR per study day and which experimental group these animals belonged to. These tests were run on each dependent variable per region.

### 2.7. Effect of Injury Group

Two-way ANOVAs for the experimental group (sham, single, and repeated) and study day (pre-injury and 1, 4, and 7 days post-injury) were conducted for absolute power and relative alpha, beta, delta, and theta power. A Levene’s test for equal variance was completed for each dependent variable. Post hoc analyses included 1-way ANOVAs with Bonferroni corrections for multiple comparisons (*p* < 0.05). All statistical tests were stratified by region of interest (frontal, left, and right temporal).

## 3. Results

**Figure 2:**
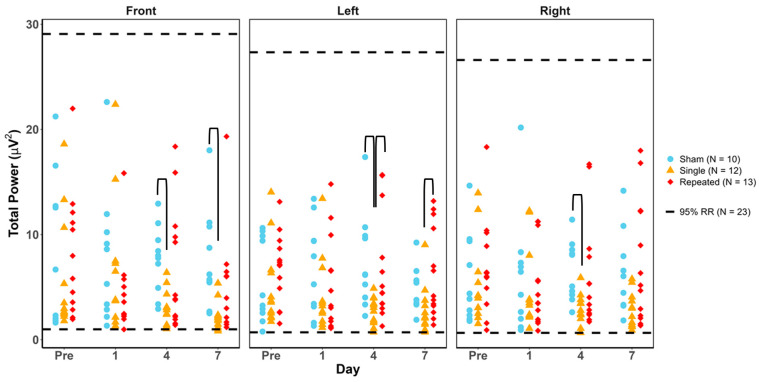
Total power values for each animal group by day and region. Blue = sham, yellow = single RNR, red = repeated RNR. The 95%ile reference ranges (RRs) are demarcated with hashed lines. No experimental animals were significantly outside of the healthy RR (*p* < 0.05). Significant Kruskal–Wallis H tests with adjusted Bonferroni corrections illustrate between-group differences (black brackets, *p* < 0.05).

**Figure 3:**
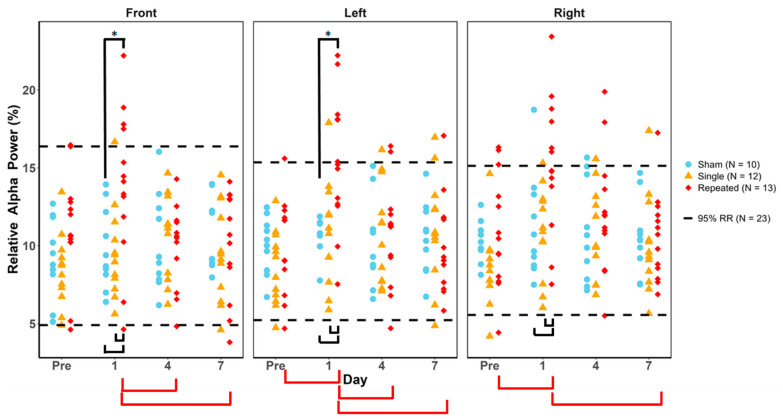
Relative alpha power values for each animal group by day and region. Blue = sham, yellow = single RNR, red = repeated RNR. The 95%ile reference ranges (RRs) are demarcated with hashed lines. Significant proportions of experimental animals outside of the healthy RR are illustrated with black overlaying bars (* = *p* < 0.05). rRNR was significantly increased outside the healthy RR compared to sham on day 1 in the frontal and left regions. Two-way ANOVA results showing significant differences between injury groups are illustrated with black (for group) or red (for day) underlying bars (*p* < 0.05). There was a significant effect of study day for the rRNR group, and this is illustrated with underlying red brackets (* = *p* < 0.05).

**Figure 4:**
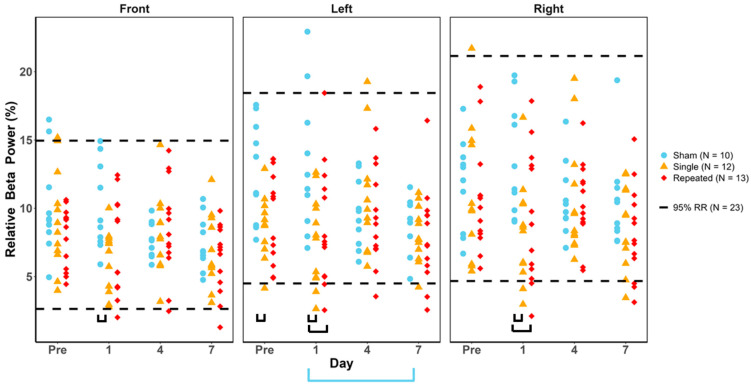
Relative beta power values for each animal group by day and region. Blue = sham, yellow = single RNR, red = repeated RNR. The 95%ile reference ranges (RRs) are demarcated with hashed lines. No experimental animals were significantly outside of the healthy RR (*p* < 0.05). Two-way ANOVA results showing significant differences between injury groups are illustrated with black or blue underlying brackets (*p* < 0.05). There was a significant effect of study day for the sham group, wherein days 1 and 7 are different from each other (blue bracket, *p* < 0.05).

**Figure 5:**
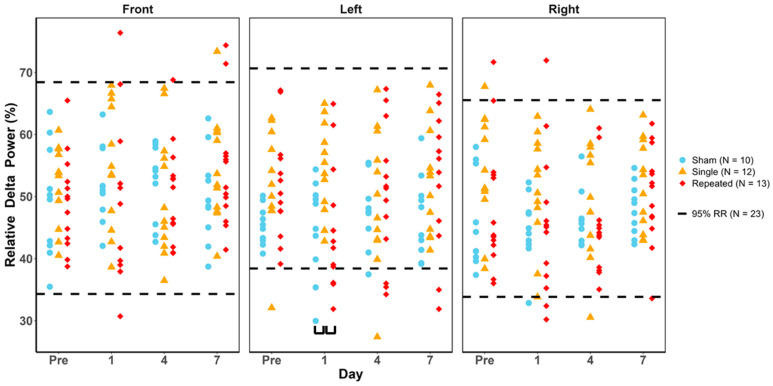
Relative delta power values for each animal group by day and region. Blue = sham, yellow = single RNR, red = repeated RNR. The 95%ile reference ranges (RRs) are demarcated with hashed lines. No injury groups were significantly outside the healthy RR (*p* < 0.05). Two-way ANOVA results showing significant differences between injury groups are illustrated with black underlying brackets (*p* < 0.05).

**Figure 6:**
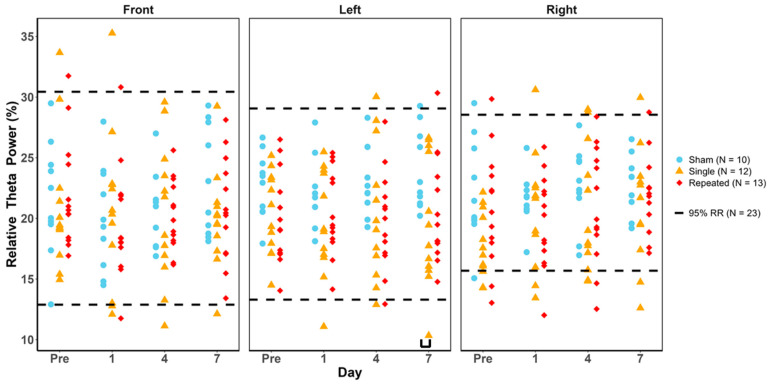
Relative theta power values for each animal group by day and region. Blue = sham, yellow = single RNR, red = repeated RNR. The 95%ile reference ranges (RRs) are demarcated with hashed lines. No injury groups were significantly outside the healthy RR (*p* < 0.05). Two-way ANOVA results showing significant differences between injury groups are illustrated with black underlying brackets, with sham having greater left temporal theta power than sRNR on day 7 (*p* < 0.05).

Total power and relative alpha, beta, delta, and theta power for sham, sRNR, and rRNR animals compared to the established healthy reference ranges are shown in Figure 2, Figure 3, Figure 4, Figure 5 and Figure 6. For each type of EEG power, statistical comparisons are presented that evaluate the proportion of experimental animals outside the healthy RRs. Table 4, Table 5, Table 6, Table 7 and Table 8 summarize the mean and standard error of the mean for each injury group, study day, and region. Two-way ANOVAs examining the effect of injury group and study day are presented next. SPSS Statistics (IBM, Armonk, NY, USA) was used for all statistical analyses reported, and the R package ggplot2 was used for all data visualizations [33]. All the statistical comparisons in what follows passed Levene’s test for equal variance except total power. For this variable, the Kruskal–Wallis H test was completed for the effect of injury group per study day and region, with adjusted Bonferroni corrections reported for each significant comparison.

### 3.1. Healthy Reference Ranges

We established healthy RRs using a large dataset of healthy animals (N = 23) and validated rs-EEG parameters with separate animals to identify that total power and relative alpha, beta, delta, and theta power are consistent measures in the healthy piglet brain in the frontal, left, and right temporal regions. These data can serve as references for other swine models in the veterinary or biomedical sciences. Validation animals scored ≥ 79% in all measures (total and relative power) in all regions except the occipital. These results indicate that these measures are consistent and that these RRs are good representations of the levels of rs-EEG activity in the healthy 4-week-old piglet. Located behind the ears and toward the neck and with thicker skull and musculature, only the occipital region was not reliably consistent for rs-EEG measurement.

### 3.2. Total Power

For total power, 100% of animals in the sham, sRNR, and rRNR groups were found to be within the healthy RR except in the frontal region, where rRNR animals scored 91% and sRNR 82% on days 1 and 7, respectively. Using the Fisher’s Exact and Cochran’s Q tests, the proportion of injury animals outside the total power healthy RR was not significant for any injury group or any day (Figure 2). When considering unequal variance between groups, using the Kruskal–Wallis H test and Bonferroni adjustments for the injury group (Figure 2 and Table 4), the results show that sRNR was significantly lower than sham at day 4 (all regions) and day 7 (frontal), and rRNR at days 4 and 7 in the left temporal region (see black brackets in Figure 2).

**Table 4:** Total power of the EEG signal for each region and injury group for pre-injury and D1, D4, and D7 post-injury. Values are mean ± SEM.

Frequency Band	Region	Group	Pre	D1	D4	D7
		Sham	7.96 ± 2.3	7.76 ± 2.02	7.62 ± 1.01 *	7.68 ± 1.48 *
	Front	sRNR	5.72 ± 1.58	6.45 ± 1.83	3.06 ± 0.49 *	2.23 ± 0.4 *
		rRNR	7.76 ± 1.61	4.6 ± 1.13	6.56 ± 1.59	5.31 ± 1.43
		Sham	5.57 ± 1.26	6.75 ± 1.4	7.49 ± 1.43 *	4.98 ± 0.68
Total Power	Left	sRNR	5.05 ± 1.12	4.35 ± 1.06	2.7 ± 0.37 *	2.75 ± 0.67 *
		rRNR	6.8 ± 0.92	5.21 ± 1.24	6.69 ± 1.4 *	6.33 ± 1.19 *
		Sham	5.79 ± 1.35	6.03 ± 1.79	6.57 ± 0.9 *	6.17 ± 1.23
	Right	sRNR	5.23 ± 1.14	4.74 ± 1.12	2.93 ± 0.43 *	2.81 ± 0.48
		rRNR	6.89 ± 1.24	4.98 ± 1.05	6.08 ± 1.42	7.27 ± 1.62

Note: Kruskal–Wallis H test results showing significant differences between injury groups per study day and region are indicated by asterisks (*), where the bolded value is the reference group for which the significant comparison is made. For example, on day 4, sRNR had significantly less frontal power than sham. Significance was accepted at *p* < 0.05.

### 3.3. Relative Alpha Power

For relative alpha power, there was a significant Fisher’s Exact test on day 1 with pairwise comparisons showing rRNR had a significantly greater proportion of animals outside the healthy RR than sham in the frontal (*p* = 0.046) and left temporal (*p* = 0.046) regions (Figure 3). By day 4, rRNR returned to be within the healthy RR. No other injury group significantly deviated outside the healthy RR on any other study day. Two-way ANOVA results confirm that rRNR was significantly different from sham and sRNR. Follow-up post hoc tests showed that rRNR had significantly greater relative alpha power on day 1 than sham and sRNR, and this increase was observed in all three regions (Table 5 and black brackets in Figure 3). Sham and sRNR were not statistically different from each other on any study day or region. Within rRNR, day 1 had significantly elevated relative alpha power than pre-injury (left and right temporal), day 4 (frontal and left temporal), and day 7 (all regions), as shown in Figure 3 with the red brackets. In summary, relative alpha power acutely increased after rRNR but not after sRNR.

**Table 5:** Relative alpha power for each region and injury group for pre-injury and D1, D4, and D7 post-injury. Values are mean ± SEM.

Frequency Band	Region	Group	Pre	D1	D4	D7
		Sham	9.23 ± 0.82	9.82 ± 0.82 *	10.16 ± 0.97	10.41 ± 0.76
	Front	sRNR	8.4 ± 0.67	9.56 ± 0.86 *	10.74 ± 0.8	9.52 ± 0.9
		rRNR	11.15 ± 0.96	13.84 ± 1.35 *	9.98 ± 0.75 †	10.15 ± 0.94 †
		Sham	10.02 ± 0.58	10.68 ± 0.39 *	10.26 ± 0.88	10.5 ± 0.73
Relative Alpha	Left	sRNR	8.56 ± 0.69	10.8 ± 0.98 *	10.78 ± 0.93	11.05 ± 1.01
		rRNR	9.91 ± 0.84 †	15.38 ± 1.19 *	10.74 ± 0.92 †	10.06 ± 0.86 †
		Sham	10.25 ± 0.41	11.21 ± 1.06 *	10.85 ± 1.02	10.55 ± 0.75
	Right	sRNR	8.55 ± 0.71	10.88 ± 0.84 *	10.74 ± 0.82	10.08 ± 0.91
		rRNR	10.88 ± 1.01 †	15.18 ± 1.21 *	12.1 ± 1.07	10.59 ± 0.78 †

Note: significant differences between injury groups per study day and region are indicated by asterisks (*), where the bolded value is the reference group for which the significant comparison is made. For example, on day 1, rRNR was greater than sham and sRNR in the frontal region. Within the rRNR group, there was an effect of study day, where † denotes a significant difference from D1. For example, frontal alpha power was decreased on days 4 and 7, in comparison to day 1.

### 3.4. Relative Beta Power

Relative beta power for all injury groups remained within the healthy reference ranges with no significant group or day effects, as the Fisher’s Exact and Cochran’s Q tests did not yield significant results (Figure 4). Two-way ANOVAs for the injury group and day followed by post hoc tests reveal that sRNR had significantly decreased frontal relative beta power than sham on day 1 (Table 6). Furthermore, the left temporal region showed significant decreases for sRNR in comparison to sham at pre-injury, but not rRNR. On day 1, sRNR and RNR had significantly decreased left and right temporal beta power compared to sham (as shown by black brackets in Figure 4).

**Table 6:** Relative beta power for each region and injury group for pre-injury and D1, D4, and D7 post-injury. Values are mean ± SEM.

Frequency Band	Region	Group	Pre	D1	D4	D7
		Sham	10.11 ± 1.13	10.02 ± 1.01 *	7.81 ± 0.42	7.43 ± 0.62
	Front	sRNR	9.14 ± 1.05	6.23 ± 0.66 *	8.18 ± 0.87	7.09 ± 0.78
		rRNR	7.82 ± 0.61	7.07 ± 0.99	8.47 ± 0.98	6.29 ± 0.7
		Sham	12.66 ± 1.17 *	13.18 ± 1.62 *	9.6 ± 0.8	8.75 ± 0.66 †
Relative Beta	Left	sRNR	8.65 ± 0.68 *	7.65 ± 0.96 *	10.44 ± 1.22	8.04 ± 0.59
		rRNR	9.46 ± 0.92	8.74 ± 1.22 *	9.28 ± 0.98	7.92 ± 0.99
		Sham	11.78 ± 1.06	13.55 ± 1.3 *	10.65 ± 0.85	10.49 ± 1.1
	Right	sRNR	10.65 ± 1.49	8.24 ± 1.08 *	10.6 ± 1.24	8.59 ± 0.87
		rRNR	10.62 ± 1.11	9.35 ± 1.35 *	10.41 ± 0.82	8.22 ± 0.95

Note: significant differences between injury groups per study day and region are indicated by asterisks (*), where the bolded value is the reference group for which the significant comparison is made. For example, sham had greater frontal beta power than sRNR on day 1. Within sham, there was an effect of the study day, where † denotes a significant difference between day 7 and day 1 (bold) in the left temporal region. Significance was accepted at *p* < 0.05.

### 3.5. Relative Delta Power

Similar to relative beta power, no injury groups were significantly outside the healthy RR, as confirmed by the Fisher’s Exact and Cochran’s Q tests (Figure 5). Therefore, all injury groups remained in the relative delta power healthy RR for all study days and regions. A two-way ANOVA (injury group and study day) followed by post hoc tests show that sRNR had a significant elevation in left temporal relative delta power on day 1 (Table 7 and black brackets in Figure 5).

**Table 7:** Relative delta power for each region and injury group for pre-injury and D1, D4, and D7 post-injury. Values are mean ± SEM.

Frequency Band	Region	Group	Pre	D1	D4	D7
		Sham	49.4 ± 2.89	51.98 ± 1.98	52.14 ± 1.92	49.99 ± 2.33
	Front	sRNR	51.84 ± 1.85	54.9 ± 2.89	50.37 ± 2.9	54.58 ± 2.53
		rRNR	49.11 ± 2.1	48.83 ± 3.63	50.5 ± 2.25	54.15 ± 2.66
		Sham	45.31 ± 0.97	44.89 ± 2.47 *	47.43 ± 1.75	46.73 ± 2.08
Relative Delta	Left	sRNR	51.92 ± 2.45	54.38 ± 2.11 *	49.49 ± 3.18	52.08 ± 2.49
		rRNR	51.72 ± 2.38	44.98 ± 2.77 *	50.22 ± 3.07	52.79 ± 3
		Sham	45.92 ± 2.42	44.68 ± 1.74	46.4 ± 1.38	47.99 ± 1.32
	Right	sRNR	54.06 ± 2.61	51.05 ± 2.56	49.46 ± 2.75	51.79 ± 2.06
		rRNR	48.4 ± 2.98	45.57 ± 3.27	44.84 ± 2.23	50.28 ± 2.16

Note: significant differences between injury groups per study day and region are indicated by asterisks (*), where the bolded value is the reference group for which the significant comparison is made. In the left temporal region, sRNR had greater delta power than sham and rRNR on day 1. Significance was accepted at *p* < 0.05.

### 3.6. Relative Theta Power

Despite the greater variability in scores for relative theta power, there were no statistically significant deviations outside the healthy RR for the injury group, study day, or region as determined by the Fisher’s Exact and Cochran’s Q tests (Figure 6). Two-way ANOVAs (injury group and study day) and post hoc analyses indicated that day 7 had significantly lower values for sRNR than sham in only the left temporal region (Table 8 and black bracket in Figure 6).

**Table 8:** Relative theta power for each region and injury group for pre-injury and D1, D4, and D7 post-injury. Values are mean ± SEM.

Frequency Band	Region	Group	Pre	D1	D4	D7
		Sham	21.62 ± 1.5	20.05 ± 1.38	20.34 ± 1.01	22.99 ± 1.43
	Front	sRNR	20.97 ± 1.6	20.56 ± 1.89	20.37 ± 1.67	19.92 ± 1.18
		rRNR	21.99 ± 1.24	19.62 ± 1.31	20.24 ± 0.8	20.72 ± 1.2
		Sham	22.73 ± 0.86	21.7 ± 0.98	23.08 ± 0.9	23.98 ± 1.04 *
Relative Theta	Left	sRNR	20.36 ± 0.97	19.57 ± 1.26	20.44 ± 1.62	19.7 ± 1.55 *
		rRNR	19.99 ± 1.05	20.47 ± 0.98	19.82 ± 1.13	20.73 ± 1.23
		Sham	22.17 ± 1.36	21.49 ± 0.68	22.61 ± 1.05	22.42 ± 0.83
	Right	sRNR	17.81 ± 0.79	20.67 ± 1.38	20.63 ± 1.51	21.2 ± 1.39
		rRNR	20.73 ± 1.33	19.75 ± 1.07	21.01 ± 1.32	21.82 ± 0.9

Note: significant differences between injury groups per study day and region are indicated by asterisks (*), where the bolded value is the reference group for which the significant comparison is made. Sham had significantly greater left temporal theta power than sRNR on day 7. Significance was accepted at *p* < 0.05.

## 4. Discussion

This large experimental study sought to evaluate the effects of very mild single and repeated head rotations scaled from heading loads that are typical in a single soccer match on rs-EEG measures and to examine their effects in the acute time frame (within 7 days after injury). We report healthy porcine reference ranges (RRs) for the first time and validated them with an independent dataset. The kinematic loads reported in this study (Table 1) reflect the very mild nature of these levels expected in typical soccer headers. An evaluation of the single and repeated rotation experimental injury groups in this study against the healthy RRs showed that relative alpha power was the only measurement variable that had an injury group (rRNR) significantly deviate outside of the RR. Specifically, the rRNR group had a significantly and transient higher proportion of animals above the RR at day 1 post-injury than the sham group. Deviation for the rRNR group returned back to a healthy RR at days 4 and 7. No significant RR findings were observed for the sRNR group or on any other study day.

**Figure 7:**
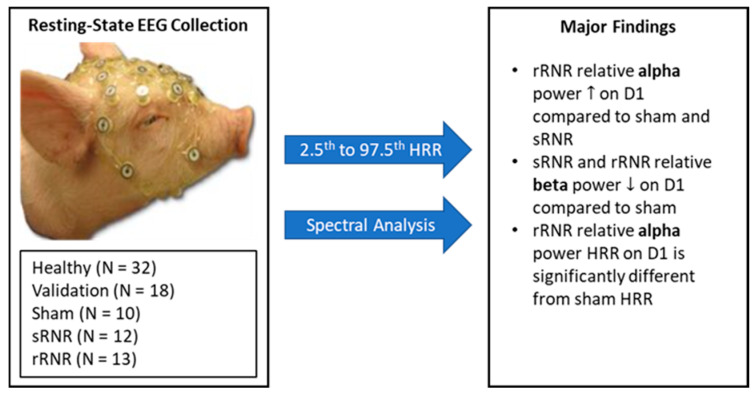
Summarization of the major findings of increases (↑) and decreases (↓) for single and repeated non-impact head rotations on resting-state EEG frequency band relative powers.

The comparisons of experimental groups and study days revealed interesting findings, where rRNR piglets had an increase in relative alpha power on day 1 post-injury over other groups. This increase was consistently observed across all regions (frontal, left, and right temporal). Within rRNR, day 1 was increased above all other study days. By days 4 and 7, relative alpha power returned to baseline levels and was not significantly different from pre-injury. Taken together with the healthy RR findings, this suggests that repetitive injuries initiate processes that transiently increase alpha activity in piglets. Interestingly, in our study, increased alpha activity was not observed after a single RNR, which can point toward a dose-dependent threshold relating to the number of head rotations and the initiation of pathways for increased activity (Figure 7). Furthermore, the transient effects observed in our piglets, which returned to normal levels by days 4 and 7, suggest that the day after injury may be a particularly vulnerable period as the brain has an increased reaction to the trauma (Table 5).

The human literature reports both decreases and increases in alpha power after TBI. In a recent systematic review, decreased alpha was observed up to 14 days post-TBI and even in patients in the subacute and chronic stages [20]. In contrast, in one study conducted by Mortazavi, Lucini [34], the authors sought to examine the trajectory of concussion recovery in teenagers and young adults ranging from 17 to 23 years old. These athletes were followed over four sports seasons, and rs-EEG was taken pre-injury, at 1 day after the concussion, at the return to sport (approximately 10 days post-injury), and after the season ended [34]. During data collection, an auditory oddball stimulus was played in the background while continuous EEG was captured. The authors noted that background unattended auditory stimuli did not affect resting-stage EEG measures [35]. Further, the athlete data collected during the sports season was compared to data collected from a separate set of subjects (N = 63) suffering from persistent post-concussion syndrome (PPCS) lasting at least 1 month that were taken on average 61 days after the injury event. There were no significant differences in peak alpha on day 1 after the concussion, at the return to sport (10 days post-injury), or at the end of the season, in comparison to pre-injury levels. The authors did find that peak alpha from another group of PPCS subjects was significantly increased compared to the pre-injury baselines and to a separate healthy reference group. The authors proposed that increased alpha activity in the PPCS group could be due to healing processes and the brain’s attempt at recovery [34].

Relative beta, delta, and theta power in our experimental groups remained in the healthy reference range. In human TBI using magnetoencephalography (MEG), beta power was found to be decreased in the frontal and temporal cortices after injury in a group of men (28 ± 5 years) in comparison to sex- and age-matched controls [36]. In the human literature, this finding holds despite the diverse mechanisms of injury (sport, motor vehicle) contributing to these results [36], whereas our precisely controlled biomechanical piglet model limited the direction of head motion to the sagittal plane.

Relative delta and theta power had comparatively fewer findings in our piglets. The healthy RR comparisons, in combination with the univariate results, suggest that these parameters may not be as sensitive to RNR injury as alpha power. Changes to delta power after a concussion are common in the human literature; however, they have been reported in a time period that is considerably beyond the 7-day period of the present study. For example, increased delta power was reported in high school athletes who suffered a concussion and returned to play within 1 month [37]. The authors noted that alterations in rs-EEG may persist despite symptom resolution. In a chronic study, higher delta power was found in military service members and veterans who reported poorer cognitive function as a result of mild TBI up to 11 years prior [38].

There are several limitations in our study. First, the findings reported in this study are specific to 4-week-old female Yorkshire piglets. Second, while male piglets were not used in the experimental study (two animals were used for validation purposes), the human literature has demonstrated sex differences in sports-related concussion outcomes, with females often reporting sustaining more severe injuries than their male counterparts, a greater number of symptoms, and poorer prognoses [39,40]. All group sizes used in this study are based upon power analyses of our previous studies of behavior outcomes for the last 15 years and are not specific to EEG outcomes. Typically, 10 to 15 animals per study group ensured that we had a clinically relevant injury effect size for behavioral studies in balance, actigraphy, event-related potentials, and biomarker outcomes. Future work in our piglets should include male piglets to observe if rs-EEG trends and trajectories are sex-specific. Additionally, rs-EEG changes between eyes-opened or eyes-closed trials are well described in the human literature, with increased alpha activity during eyes-closed [19,37]. The rs-EEG was collected in the awake animals in our study; however, there were some animals, particularly the injured animals, that were prone to closing their eyes after RNR. Care was taken to observe when an animal’s eyes were closed to prompt research staff to gently scratch the animal’s body or rub the snout to wake them up before data collection was initiated; however, there were instances when the animal would close their eyes part way through the 1-min data acquisition trial.

While these behavioral aspects were not recorded in our study, they highlight the need for future studies designed to capture physiological and behavioral measures along with rs-EEG to inform impairments across multiple domains after TBI. In addition to cognition, human TBI is also known to affect motor behavior (gait, balance, reaction time), activity levels, and sleep and is associated with light and sound sensitivities, headaches, and dizziness [41,42,43]. The diverse injury mechanisms (falls, collisions, and direction) in human TBI contribute to the myriads of deficits and symptoms reported in patients [44]; however, relationships between head injury causes and outcomes (symptoms, behavior) can be better elucidated in the human literature by stratifying by biomechanical causes of injury and establishing relationships between the types and time courses of signs and symptoms.

## 5. Conclusions

We present the first healthy reference ranges for resting-state EEG measures for awake piglets and evaluate a separate cohort of experimental piglets subjected to single and repeated rapid head rotations against these established values. Healthy RRs can provide a point of reference for other swine models in the veterinary or biomedical sciences. Furthermore, in our experimental animals, total power for sham, sRNR, and rRNR groups did not significantly deviate from the healthy RR; however, there were regional variations in sustained decreases at 4 and 7 days post-injury for the sRNR group. We report a significant but transient increase in relative alpha power after repeated rapid non-impact head rotations at day 1 post-injury. This finding was substantiated by comparisons with healthy RRs; the rRNR group had significant values above the RR compared with the sham group on day 1. Within the healthy reference range, we observed a decrease in relative beta power after both single and repeated RNR relative to sham on day 1, suggesting that the day after injury may be a particularly vulnerable period as the brain is responding to trauma. We found fewer significant findings for relative delta and theta power in our piglets in the acute time period, and they may not be as sensitive to RNR injury in comparison to alpha and beta power. Future studies should include male subjects and a longer study duration to investigate and inform clinical prognoses from rs-EEG measurements.

## Figures and Tables

**Table 1:** Mean ± standard error of the mean of angular velocity and acceleration for sRNR and rRNR animal cohorts.

Injury Cohort	Rotation Intensity	Angular Velocity (rad/s)	$\mathrm{Angular}\;\mathrm{Acceleration}\;(\mathrm{rad}/s^{2})$
sRNR	High	103.87 ± 0.49	36,586.56 ± 1280.56
rRNR	High	104.61 ± 0.41	38,424.99 ± 544.55
	Medium	61.30 ± 0.21	14,852.46 ± 200.57

**Table 2:** Healthy reference ranges for total and relative power reporting the 2.5th, 50th, and 97.5th percentile values.

Frequency Band	Region	2.5th	50th	97.5th
Total Power	Frontal	0.9903625	5.3535	29.07608
	Left	0.7045	4.92495	27.33649
	Right	0.65306	4.1834	26.59986
	Occipital	0.5282725	2.21575	12.54098
Relative Alpha	Frontal	4.91397	9.5276	16.38051
	Left	5.2316	10.1217	15.35884
	Right	5.56507	9.9164	15.13626
	Occipital	5.033005	9.74	17.6559
Relative Beta	Frontal	2.63779	6.8624	14.95164
	Left	4.50008	9.3918	18.44081
	Right	4.67446	9.6784	21.15121
	Occipital	2.32121	10.5398	20.0747
Relative Delta	Frontal	34.3137	52.0001	68.43408
	Left	38.42119	48.4386	70.65963
	Right	33.84997	47.1953	65.53695
	Occipital	30.95841	48.9943	67.29821
Relative Theta	Frontal	12.88577	20.0088	30.44262
	Left	13.2979	21.595	29.0647
	Right	15.68316	20.9958	28.54675
	Occipital	12.2019	20.7489	33.00333

## Data Availability

The data presented in this study are available on request from the corresponding author due to privacy restrictions.
